# In-hospital mortality associated with the misdiagnosis or unidentified site of infection at admission

**DOI:** 10.1186/s13054-019-2475-9

**Published:** 2019-06-06

**Authors:** Toshikazu Abe, Yasuharu Tokuda, Atsushi Shiraishi, Seitaro Fujishima, Toshihiko Mayumi, Takehiro Sugiyama, Gautam A. Deshpande, Yasukazu Shiino, Toru Hifumi, Yasuhiro Otomo, Kohji Okamoto, Joji Kotani, Yuichiro Sakamoto, Junichi Sasaki, Shin-ichiro Shiraishi, Kiyotsugu Takuma, Akiyoshi Hagiwara, Kazuma Yamakawa, Naoshi Takeyama, Satoshi Gando, for the JAAM SPICE Study Group, Satoshi Gando, Toshikazu Abe, Kohji Okamoto, Seitaro Fujishima, Junichi Sasaki, Yasukazu Shiino, Yasuhiro Otomo, Shin-ichiro Shiraishi, Kiyotsugu Takuma, Toru Hifumi, Kazuma Yamakawa, Toshihiko Mayumi, Atsushi Shiraishi, Akiyoshi Hagiwara, Takashi Muroya, Kaoru Koike, Hideaki Anan, Manabu Sugita, Yasuo Miki, Hisashi Yamashita, Hirotada Kittaka, Junichi Maehara, Sho Nachi, Kazuma Morino, Atsumi Hoshino, Hiroyuki Yamaguchi, Masahiro Harada, Hiroyasu Ishikura, Masato Kawakami, Yoshizumi Deguchi, Hideaki Yoshihara, Yoshihiro Hanaki, Kunihiko Okada, Tadashi Kaneko, Kazuya Kiyota, Yoshihiro Shimizu

**Affiliations:** 1 Department of General Medicine, Juntendo University, 2-1-1, Hongo, Bunkyo-ku, Tokyo, 113-0033 Japan; 2 Department of Health Services Research, Faculty of Medicine, University of Tsukuba, Tsukuba, Japan; 3 Health Services Research and Development Center, University of Tsukuba, Tsukuba, Japan; 4 Department of Medicine, Muribushi Project for Okinawa Residency Programs, Okinawa, Japan; 5 Emergency and Trauma Center, Kameda Medical Center, Kamogawa, Japan; 6 Center for General Medicine Education, Keio University School of Medicine, Tokyo, Japan; 7 Department of Emergency Medicine, School of Medicine, University of Occupational and Environmental Health, Kitakyushu, Japan; 8 Diabetes and Metabolism Information Center, Research Institute, National Center for Global Health and Medicine, Tokyo, Japan; 9 Department of Public Health, Graduate School of Medicine, The University of Tokyo, Tokyo, Japan; 10 Department of Acute Medicine, Kawasaki Medical School, Kurashiki, Japan; 11 Department of Emergency and Critical Care Medicine, St. Luke’s International Hospital, Tokyo, Japan; 12 Trauma and Acute Critical Care Center, Medical Hospital, Tokyo Medical and Dental University, Tokyo, Japan; 13 Department of Surgery, Center for Gastroenterology and Liver Disease, Kitakyushu City Yahata Hospital, Kitakyushu, Japan; 14 Department of Disaster and Emergency Medicine, Kobe University Graduate School of Medicine, Kobe, Japan; 15 Emergency and Critical Care Medicine, Saga University Hospital, Saga, Japan; 16 Department of Emergency and Critical Care Medicine, Keio University School of Medicine, Tokyo, Japan; 17 Department of Emergency and Critical Care Medicine, Aizu Chuo Hospital, Aizuwakamatsu, Japan; 18 Emergency & Critical Care Center, Kawasaki Municipal Kawasaki Hospital, Kawasaki, Japan; 19 Department of Emergency Medicine, Niizashiki Chuo General Hospital, Niiza, Japan; 20 Division of Trauma and Surgical Critical Care, Osaka General Medical Center, Osaka, Japan; 21 Advanced Critical Care Center, Aichi Medical University Hospital, Nagakute, Japan; 22 Division of Acute and Critical Care Medicine, Hokkaido University Graduate School of Medicine, Sapporo, Japan; 23 Department of Acute and Critical Care Medicine, Sapporo Higashi Tokushukai Hospital, Sapporo, Japan

**Keywords:** Diagnosis, Sepsis, Infection, Source

## Abstract

**Background:**

Rapid detection, early resuscitation, and appropriate antibiotic use are crucial for sepsis care. Accurate identification of the site of infection may facilitate a timely provision of appropriate care. We aimed to investigate the relationship between misdiagnosis of the site of infection at initial examination and in-hospital mortality.

**Methods:**

This was a secondary-multicenter prospective cohort study involving 37 emergency departments. Consecutive adult patients with infection from December 2017 to February 2018 were included. Misdiagnosis of the site of infection was defined as a discrepancy between the suspected site of infection at initial examination and that at final diagnosis, including those infections remaining unidentified during hospital admission, whereas correct diagnosis was defined as site concordance. In-hospital mortality was compared between those misdiagnosed and those correctly diagnosed.

**Results:**

Of 974 patients included in the analysis, 11.6% were misdiagnosed. Patients diagnosed with lung, intra-abdominal, urinary, soft tissue, and CNS infection at the initial examination, 4.2%, 3.8%, 13.6%, 10.9%, and 58.3% respectively, turned out to have an infection at a different site. In-hospital mortality occurred in 15%. In both generalized estimating equation (GEE) and propensity score-matched models, misdiagnosed patients exhibited higher mortality despite adjustment for patient background, site infection, and severity. The adjusted odds ratios (misdiagnosis vs. correct diagnosis) for in-hospital mortality were 2.66 (95% CI, 1.45–4.89) in the GEE model and 3.03 (95% CI, 1.24–7.38) in the propensity score-matched model. The difference in the absolute risk in the GEE model was 0.11 (0.04–0.18).

**Conclusions:**

Among patients with infection, misdiagnosed site of infection is associated with a > 10% increase in in-hospital mortality.

**Electronic supplementary material:**

The online version of this article (10.1186/s13054-019-2475-9) contains supplementary material, which is available to authorized users.

## Key points

- Early misdiagnosis or unidentified site of infection resulted in doubling of odds ratio of in-hospital mortality.
- Extremely time-sensitive care bundles may warrant reconsideration, renewing focus on enhancing the precision of diagnosis and subsequent treatment.

## Introduction

Sepsis is one of the most life-threatening and resource-intensive conditions encountered in hospital care across the globe [1]. Rapid identification followed by the initiation of early resuscitation and swift administration of appropriate antibiotics is crucial before the body’s immune system is overwhelmed [2]. The Surviving Sepsis Campaign guidelines call for immediate resuscitation and management of sepsis, and it defines sepsis as an emergent disease similar to trauma, heart attack, and stroke [3]. However, sepsis is characterized by various etiologies and pathophysiological conditions, thereby making it substantially more complicated to treat than many other time-sensitive emergencies. Previous studies have suggested that clinical differences of the site infection may be important in helping clinicians to appropriately stratify risk and in guiding clinical decision-making for treatment [4–6].

Currently, most sepsis patients are likely to receive recommended evidence-based care including use of bundles. However, these time-sensitive bundles were originally developed to maximize both speed and accuracy of the identification and management of sepsis [3]. In reality, the speed of care and readily quantifiable parameter are tied to financial rewards and penalties, emphasizing diagnostic accuracy. If the inaccuracies of diagnosis (misdiagnosis) lead to higher mortality or other suboptimal outcomes, time bundles may not be ideal tools to improve the quality of care in sepsis. Thus, it is important to clarify the relationship between the misdiagnosis of the site of infection and adverse events in sepsis care. The aim of the present study was to investigate the effect of misdiagnosed site of infection at the initial examinations of patients’ outcomes.

## Methods

### Design, setting, and participants

The present study focuses on a secondary analysis of an emergency room (ER) subset of the Japanese Association for Acute Medicine Sepsis Prognostication in Intensive Care Unit and Emergency Room (JAAM SPICE-ER) [7]. The present multicenter, prospective cohort study included 37 emergency departments from December 2017 to February 2018. Adult patients (≥ 16 years) were consecutively included in the study if they had suspected infection as defined by the receipt of any kind of antibiotic, needed to obtain a culture of body fluids or imaging required to identify an infectious focus. Participants were to be hospitalized in one of the study hospitals or had died in the emergency department (ED). Exclusion criteria included those patients not hospitalized or transferred to a non-study hospital. For this post hoc analysis, we excluded patients free of an infectious disease following their final diagnoses list at discharge.

### Data collection

Data were extracted from the SPICE database, which was compiled by SPICE investigators. Collected variables included relevant patient information, such as demographics, comorbidities, the degree of clinical frailty, vital signs, and suspected site of infection at initial examination and at final diagnosis. In-hospital mortality was recorded as the primary outcome. Secondary outcomes were ventilator-free days (VFD), intensive care unit-free days (ICU-free days), length of hospital stay (LOS), and disposition at discharge. Data collection was performed as part of the clinical routine workup. Data were recorded by SPICE site investigators throughout the patient’s hospital stays. If data were found to be missing, the SPICE committee requested a reconfirmation of data extraction from SPICE investigators.

### Data definitions

Infection sites included 12 foci: lung, intra-abdominal, urinary tract, soft tissue, the central nervous system (CNS), osteoarticular, endocardium, wound, catheter-related, implant device-related, other, or unidentified infections. The diagnosis at the infection site was recorded at the initial examination in the ER (initial diagnosis) and recorded at discharge (final diagnosis). The initial diagnosis was defined as the most suspected site of infection at the initial examination, and the final diagnosis was defined as the main site of infection at the final diagnosis. Misdiagnosis of the site of infection was defined as either discrepancy between initial and final diagnoses or infection that occurred at the unidentified site. A correct diagnosis was defined as a diagnosis in accordance with the infection site between initial and final diagnoses. VFD was defined as the number of days within the first 28 days after admission during which a patient was able to breathe without a ventilator. The VFD of patients who died during the study period was set as 0. ICU-free days were calculated like the way VFD was calculated. Disposition at discharge was categorized as home, transfer to another facility (including long-term care and nursing homes), or death.

### Analysis

Descriptive statistics included counts (proportions) for categorical variables. Median (interquartile range, IQR) is categorized as continuous variables as many variables did not exhibit a normal distribution. Given a low rate of missing data of 0.8%, no imputation was made for the missing data.

We first compared the patients’ baseline characteristics and demographic data, clinical background, vital signs, and outcomes associated with misdiagnosis or unidentified site of infection versus correct initial diagnosis at the infection site. Additionally, we assessed the rates of misdiagnosis by the infection site. In addition, we constructed a multivariable model to adjust for potential confounding factors and specified a GEE with an exchangeable working correlation matrix to account for clustering by hospital. Age, Charlson comorbidity index (CCI), quick sepsis-related organ failure assessment (qSOFA) score, and infection site at final diagnosis (lung, intra-abdominal, urinary tract, soft tissue, rare, and unidentified) were used for adjustment; covariates were chosen a priori based on previous reports [4, 5] and clinical importance. Based on probability determined from the GEE model, we subsequently used marginal standardization [8] to estimate absolute differences in the risk of in-hospital mortality due to misdiagnosis or unidentified site of infection. A subgroup analysis focusing on patients with qSOFA ≥ 2 was performed to explore the clinical question whether in-hospital mortality is associated with misdiagnosis or unidentified site of infection at admission among more severely ill patients.

We also developed a propensity score-matched (PSM) model to pursue further evidence for a causal relationship between misdiagnosis or unidentified site of infection and mortality. The propensity score for misdiagnosis or unidentified site of infection was determined using a logistic regression with the below-listed covariates as independent variables: age, CCI, clinical frailty scale, mean blood pressure, heart rate, respiratory rate, Glasgow coma scale (GCS) score, and infection site at final diagnosis. Infections at final diagnosis were categorized as lung, intra-abdominal, urinary tract, soft tissue, rare (CNS; osteoarticular; endocardium; wound; catheter-related; and implant device-related), other, and unidentified. Propensity score matching extracted 1:1 ratio matched pairs of subjects with a misdiagnosis, including unidentified or correct diagnosis, based on the average propensity score with a caliper (0.2). The absolute standardized difference of variables for the PS estimation was used to assess the match balance. An absolute standardized difference of less than 0.1 was considered as an acceptable match balance between the groups.

We performed the sensitivity analysis, as described in the GEE models for all patients and patients with qSOFA ≥ 2, and PSM model, excluding those with an unidentified site of infection. All *p* values were two-sided. The *p* values less than 0.05 were considered statistically significant. Statistical analyses were performed with Stata software, version 15.1 (StataCorp, College Station, TX, USA).

## Results

A total of 1060 patients with suspected infection were included during the study period. In total, 86 patients, i.e., 81 patients identified as ultimately not having an infection and 5 with missing data from the infection site, were excluded from the study. Of the remaining 974 participants, 113 patients (11.6%) had misdiagnosed initial site of infection: 37 patients (32.7%) with unidentified site of infection, and 76 patients (67.3%) whose initial suspected site of infection was incorrect. Their median age was 78 (IQR 68–85), and 60.4% were male. Baseline characteristics were not found to differ substantially between patients with misdiagnosis or with unidentified site of infection and those with correct diagnosis (Table 1; Additional file 1: Table S1). However, those in the misdiagnosis group had a lower GCS score and lower partial pressure of arterial carbon dioxide (PCO_2_). Table 2 presents the misdiagnosis rates by the site of infection. Patients diagnosed with lung, intra-abdominal, urinary, and soft tissue infection at initial examination, 4.2%, 3.8%, 13.6%, and 10.9% respectively, turned out to have an infection at a different site. Patients diagnosed with rare sites of infection (CNS, osteoarticular, endocardium, wound, catheter-related, implanted device-related, and others) at initial examination, with various rates (58.3%, 0%, 16.7%, 25.0%, 33.3%, 50.0%, and 25.5%) respectively, turned out to have infection at a different site (Table 2; Additional file 1: Table S1).

**Table 1:** Characteristics of patients with infection comparing misdiagnosis or unidentified with the correct diagnosis at site of infection (*n* = 974)

Characteristics	Misdiagnosed or unidentified site of infection	Correctly diagnosed site of infection	*p* value
	113	861	
Age at admission (years old)	78 (66–85)	78 (68–85)	0.94
Sex (male)	62 (54.8)	526 (61.1)	0.20
BMI (kg/m^2^)	21.8 (19.5–24.3)	21.1 (18.4–23.9)	0.13
Charlson comorbidity index			
0	40 (35.4)	241 (28.0)	0.07
1, 2	40 (35.4)	301 (35.0)	
3, 4	22 (19.5)	152 (17.7)	
≥ 5	11 (9.7)	167 (19.4)	
Clinical frailty scale			
1, 2, 3	52 (46.0)	337 (39.1)	0.19
4	14 (12.4)	147 (17.1)	
5	6 (5.3)	90 (10.5)	
6	12 (10.6)	103 (12.0)	
≥ 7	29 (25.7)	184 (21.4)	
GCS	14 (11–15)	15 (13–15)	< 0.01
Intubated	7 (6.2)	28 (3.3)	0.11
SBP (mmHg)	128 (100–150)	125 (105–148)	0.88
DBP (mmHg)	69 (58–87)	72 (60–84)	0.84
MBP (mmHg)	89 (74–103)	90 (75–105)	0.82
HR (/min)	102 (81–118)	99 (84–113)	0.84
RR (/min)	22 (18–26)	23 (18–28)	0.92
Body temperature (°C)	37.7 (36.6–38.8)	37.6 (36.7–38.5)	0.68
qSOFA score (*n* = 951)			
0	17 (15.2)	197 (23.1)	0.08
1	42 (37.5)	333 (39.0)	
2	42 (37.5)	230 (27.0)	
3	11 (9.8)	94 (11.0)	
WBC (/μL)	10,800 (7300–15,470)	11,000 (7500–14,800)	0.96
Lactate (mmol/L)	2.1 (1.3–3.7)	1.9 (1.3–3.1)	0.10
PCO_2_ (mmHg)	34.3 (28.5–42.7)	37.6 (31.1–44)	0.02

*BMI* body mass index, *GCS* Glasgow coma scale, *SBP* systolic blood pressure, *DBP* diastolic blood pressure, *MBP* mean blood pressure, *HR* heart rate, *RR* respiratory rate, *qSOFA* quick sequential organ failure assessment

Missing: BMI = 135, qSOFA = 8, SBP = 2, DBP = 3, MBP = 3, RR = 8, body temperature = 1, lactate = 98, PCO_2_ = 101

**Table 2:** Misdiagnosis rate by the site of infection (n = 974)

Site of infection at the initial diagnosis	Misdiagnosis rate (%)	Site of infection at the final diagnosis											
	113/974 (11.6)	Lung	Intra-abdominal	Urinary tract	Soft tissue	Central nervous system	Osteo-articular	Endocardium	Wound	Catheter-related	Implant device-related	Other	Unidentified
Lung	20/474 (4.2)	454	1	8	0	0	0	0	0	1	0	3	7
Intra-abdominal	7/186 (3.8)	2	179	4	0	0	0	0	0	0	0	0	1
Urinary tract	20/147 (13.6)	6	5	127	2	0	0	0	0	1	0	1	5
Soft tissue	5/46 (10.9)	1	1	1	41	0	1	0	0	0	1	0	0
Central nervous system	7/12 (58.3)	2	1	0	0	5	0	1	0	0	0	2	1
Osteo-articular	0/6 (0)	0	0	0	0	0	6	0	0	0	0	0	0
Endocardium	1/6 (16.7)	0	0	0	0	0	0	5	0	0	0	0	1
Wound	1/4 (25.0)	0	0	0	0	0	0	0	3	0	0	0	1
Catheter-related	1/3 (33.3)	1	0	0	0	0	0	0	0	2	0	0	0
Implant device-related	1/2 (50.0)	0	1	0	0	0	0	0	0	0	1	0	0
Other	13/51 (25.5)	5	2	0	2	1	0	0	0	0	0	38	3
Unidentified*	37/37 (100)	6	2	8	2	0	1	0	0	0	0	2	16

*Patients with an infection of unidentified origin at the initial or final diagnosis were defined as being misdiagnosed

**Fig. 1:**
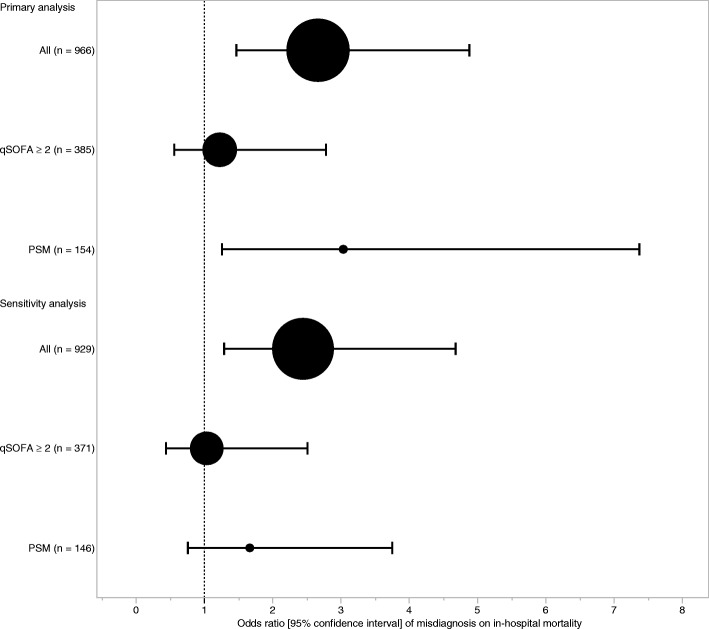
The relationship between in-hospital mortality and misdiagnosis or unidentified of the site of infection among patients with infection using the generalized estimating equations (GEE) with exchangeable working-correlation matrix models and propensity score-matched (PSM) analysis. The primary analysis described the GEE models for all patients and patients with qSOFA ≥ 2 and the PSM model. The GEE model for all patients adjusted: age, Charlson comorbidity index, clinical frailty scale, qSOFA, site of infection at final diagnosis (lung, intra-abdominal, urinary tract, soft tissue, rare [central nervous system (CNS); osteoarticular; endocardium; wound; catheter-related; and implant device-related], other, or unidentified). The GEE model for patients with qSOFA ≥ 2 adjusted: the same variables of the GEE model for all patients except qSOFA. The PSM model adjusted: age, Charlson comorbidity index, clinical frailty scale, MBP, HR, RR, GCS, site of infection at final diagnosis (lung, intra-abdominal, urinary tract, soft tissue, rare [central nervous system (CNS); osteoarticular; endocardium; wound; catheter-related; and implant device-related], other, or unidentified). The sensitivity analysis described the GEE models for all patients and patients with qSOFA ≥ 2 and PSM model excluding patients with an unidentified site of infection. The GEE model for all patients adjusted: age, Charlson comorbidity index, clinical frailty scale, qSOFA, site of infection at final diagnosis (lung, intra-abdominal, urinary tract, soft tissue, rare [central nervous system (CNS); osteoarticular; endocardium; wound; catheter-related, implant device-related, and other]). The GEE model for patients with qSOFA ≥ 2 adjusted: the same variables of the GEE model for all patients except qSOFA. The PSM adjusted: age, Charlson comorbidity index, clinical frailty scale, MBP, HR, RR, GCS, site of infection at final diagnosis (lung, intra-abdominal, urinary tract, soft tissue, rare [central nervous system (CNS); osteoarticular; endocardium; wound; catheter-related, implant device-related, and other]). qSOFA quick sequential organ failure assessment, PSM propensity score-matched

Overall in-hospital mortality rate was 15.0%. In bivariate analysis, patients with misdiagnosis or unidentified site of infection had a significant higher in-hospital mortality than those with correct diagnosis in both all-patient [28/113 (24.8%) vs. (118/861 (13.7%); *p* < 0.01] and PSM [20/77 (26.0%) vs. 8/77 (10.4%); *p* = 0.01] cohorts. However, no significant difference in in-hospital mortality among patients with qSOFA ≥ 2 was observed [16/54 (29.6%) vs. 69/331 (20.9%); *p* = 0.15] (Table 3). Those in the misdiagnosis group also stayed longer in ICU and ventilator than those in the correct diagnosis group, although LOS did not differ statistically between the groups**.** In both GEE and PSM models, patients with misdiagnosis or unidentified site of infection demonstrated higher mortality; the adjusted odds ratios for misdiagnosis or unidentified site of infection versus correct diagnosis for in-hospital mortality were 2.66 (95% CI 1.45–4.89) in the GEE model and 3.03 (95% CI 1.24–7.38) in the PSM model (Fig. 1). The difference in the absolute risk in the GEE model was 0.11 (0.04–0.18). For patients with qSOFA ≥ 2, the differences were smaller and not significant [OR 1.22 (95% CI 0.54–2.79)]. Sensitivity analyses showed similar results although the PSM model in sensitivity analyses did not show statistical significance (Fig. 1).

**Table 3:** Outcome comparison between misdiagnosis or unidentified and correct diagnosis of site of infection among patients with infection (*n* = 974)

Characteristics	Misdiagnosed or unidentified site of infection	Correctly diagnosed site of infection	*p* value
	113	861	
In-hospital mortality			
All	28 (24.8)	118 (13.7)	< 0.01
qSOFA ≥ 2 (*n* = 385)	16 (29.6)	69 (20.9)	0.15
PSM (*n* = 154)	20 (26.0)	8 (10.4)	0.01
28-day mortality	20/97 (20.6)	101/756 (13.4)	0.05
ICU-free days	27 (0–28)	28 (24–28)	< 0.01
Ventilator-free days	28 (2–28)	28 (28–28)	0.02
Length of hospital stay	15 (8–26)	14 (8–28)	0.59
Survivor disposition			
Home	64 (56.6)	517 (60.0)	< 0.01
Transfer	21 (18.6)	226 (26.2)	

*ICU* intensive care unit, *qSOFA* quick sequential organ failure assessment, *PSM* propensity score-matched

Missing data: 28-day mortality = 121, ICU-free days = 110, ventilator-free days = 45, length of hospital stay = 49

## Discussion

### Summary

The present study comprises a secondary analysis of patients with infection using a multicenter prospective cohort study in Japan. We evaluated the clinical outcomes related to misdiagnosis or unidentified site of infection. Of the 974 patients admitted with infection, 11.6% experienced misdiagnosis or unidentification regarding the site of infection. In terms of mortality among patients with infection, the odds of mortality for patients with a misclassified infection site at admission were two-fold higher than for those with an accurately identified site.

To the best of our knowledge, no studies have evaluated the impact of misdiagnosis on the outcomes of the site of infection. A cross-sectional cellulitis study showed that the misdiagnosis of lower-extremity cellulitis might lead to unnecessary patient morbidity and considerable increases in health care costs [9]. The study reported that the misdiagnosis rate of diagnosis as having cellulitis was 30.5% (79/259) as compared with that of soft-tissue infections at 10.9% (5/46) in the present study. A previous study reported that the rate of misdiagnosis of appendicitis was 33.3% (58/174) among the non-pregnant women [10], whereas the rate of misdiagnosis of intra-abdominal infection was 3.8% (7/186) in the present study. The differences may be associated to differences in study size, the patient selection as limited to those ED cases requiring hospitalization, systems-based healthcare differences by country, and temporal improvements in diagnostic technology, such as ready access to advanced imaging. Despite the smaller sample in our study, the effects of misdiagnosis or unidentified site of infection on mortality remained robust.

Our study found that, among the four major sites of infection, patients with urinary tract and soft-tissue infections were at high risk of misdiagnosis or unidentified site of infection. Patients with infection at rare sites (i.e., not at any of the four major sites of infection) also had a higher risk of misdiagnosis or unidentified site of infection. From a clinical perspective, it is somewhat reasonable that mortality is correlated with the site of infection [4, 5]. However, both the GEE and PSM models, adjusting for the site of infection and severity, demonstrated that initial misdiagnosis or unidentified site of infection was independently associated with in-hospital mortality. Misdiagnosis or unidentified site of infection play critical roles.

A previous study of intra-abdominal infections reported that misdiagnosis resulted in inadequate or delayed source control [11]. Other studies have shown that an inappropriate choice of antibiotic therapy, due to inappropriate diagnosis, has been related to poor outcomes [12, 13]. In addition, a retrospective cohort study at an academic hospital reported that vague symptoms, which were not specific to infection, were associated with delayed antibiotic administration and a higher risk of mortality [14]. As there were more patients with an altered mental status (lower GCS) in the misdiagnosis group than in the correct diagnosis group in this study, we hypothesized that an altered mental status might play a role in reportedly vague symptoms that consequent delayed the provision of appropriate care. Nonetheless, the appropriate administration of optimal antibiotic regimens is likely to play an important role in the observed favorable outcomes. Moreover, when we compared matched and unmatched for the PS model, GCS and the site of infection at the final diagnosis seemed to play a role for adjustment. Although these were confounders, they could also be indicators considered as the propensity of misdiagnosis or unidentification of the infection site.

With regard to the appropriateness of antibiotics, our previous research from Japan [15] suggests that broad-spectrum antibiotics, such as the guideline-based use of carbapenems [16], are relied on the majority of sepsis cases. Despite this assertion, recent improvements have not been observed in the outcomes of sepsis. This suggests that the sensitivity to antibiotics may not be the only relevant factor when it comes to optimal antibiotic choice as it remains necessary to carefully select antibiotics that offer higher efficacy based on other important clinical factors, including bacterial species, hospital epidemiology, site of infection, and other diseases and patient characteristics. Thus, the idea of carbapenems as de facto first-line treatment may warrant reconsideration.

Regarding patient selection, we excluded patients who were identified as ultimately not having an infection by the time of discharge. The purpose of our post hoc analysis was not to predict misdiagnosis or unidentification of the infection itself, but rather to identify the outcomes related to misdiagnosis or unidentification among patients with infection at admission. Patients without infection were excluded. Regarding the selection of covariates to control the influence on mortality of the site of infection, we chose the site of infection at final diagnosis rather than the site of infection at initial diagnosis. This is because when patients arrived at the ED, they must have already been suffering from the infection at the site found at final diagnosis even if they were classified as being a misdiagnosed site of infection at the initial stage.

For subgroup analysis, we analyzed patients with qSOFA scores of ≥ 2, more severely ill patients, who were suspected having sepsis. In this model, the coefficient point estimate of misdiagnosis or unidentified site of infection was small for the more severely subgroup as compared with that of the GEE models for all infected patients. This suggests that in-hospital mortality rate may depend more on the severity of subsequent organ failure than on infection itself, at least among more severely ill patients. This may indicate that a prompt and accurate approach to management is critical before the body’s immune system is overwhelmed. It should be noted that this subgroup was underpowered, due to the small sample size, making it difficult to identify statistically significant differences.

It is somewhat intuitive that patients with infection, including those with presumed sepsis, require quick and accurate diagnosis and treatment. However, recent sepsis care has increasingly focused on how quickly care is provisioned; our findings suggest that future studies, focusing on the trade-off between speed and accuracy, are needed, serving as a critical concept beyond the scope of the current study. A retrospective study of community-acquired pneumonia showed that time-limited antibiotic administration tied to financial compensation might lead to an inaccurate diagnosis and inappropriate utilization of antibiotics [17]. As sepsis care becomes increasingly resource-intensive, the potentially deleterious effects of sepsis-specific protocols on patients simultaneously receiving care in the ED without sepsis warrant a careful consideration, relatively to austere healthcare environments. Given that a balance between speed and accuracy is needed for optimal care, a fixed strategy for the achievement of sepsis goals may not be optimal [18].

### Limitations

This study has several important limitations that warrant discussion. First, the nature of a post hoc study precludes definitive identification of causal relationships between observed characteristics and outcomes. Second, there was a possibility of selection bias as our study only included ED patients at tertiary-level emergency care facilities. A large number of patients with infections are undoubtedly observed outside the tertiary-level facilities. However, most patients with infection progressing to sepsis are hospitalized through large and well-resourced tertiary-level EDs. Third, we categorized patients with unidentified infection sites into the misdiagnosis group. Unidentified sites of infection may have misdiagnosed or patients may not have had an infection site, such as those with primary bacteremia. To investigate the robustness of our findings considering this limitation, a sensitivity analysis, excluding those with an unidentified site of infection, was performed, which showed similar results (Fig. 1). Fifth, because our data did not include sepsis-related organ failure assessment components in their entirety, the population analyzed in this study contained patients with infection but not sepsis. Our approach was similar to that performed by Seymour et al., who performed one of the original sepsis-3 studies [16]. To test the comprehensive effect of misdiagnosis for sepsis, recruitment of patients with infection, including those with sepsis and non-sepsis, is needed. As done by Seymour [16], we too excluded patients without infection at discharge from our study population. This might have led to a selection bias; moreover, we did not include patients without infection [16] because our primary aim was to investigate the clinical importance of accurately diagnosing the infection site for selecting appropriate treatments, such as antibiotics and source controls. As noted above, an emphasis on speed may come at an expense of clinical accuracy, especially in a busy ED; the clinical impact of this trade-off remains unclear and strongly warrants further study.

## Conclusions

Among patients with infection presented to the ED, nearly a tenth was misdiagnosed at the site of infection. Early misdiagnosis or unidentification of the site of infection resulted in a more than doubling of odds ratio of in-hospital mortality rate, suggesting a need for a renewed focus on accurate diagnosis at the infection site in sepsis care.

## Additional file

Additional file 1: **Table S1.** Baseline characteristics of patients with infection in the propensity-matched model. BMI = body mass index, GCS = Glasgow coma scale, MBP = mean blood pressure, HR = heart rate, RR = respiratory rate, ref. = reference, the SD = standardized difference. Rare: central nervous system (CNS); osteoarticular; endocardium; wound; catheter-related; and implant device-related at final diagnosis. (DOCX 20 kb)

## Abbreviations

CCI: Charlson comorbidity index
CNS: Central nervous system
ED: Emergency department
ER: Emergency room
GCS: Glasgow coma scale
GEE: Generalized estimating equation
ICU-free days: Intensive care unit-free days
IQR: Interquartile range
JAAM SPICE-ER: Japanese Association for Acute Medicine Sepsis Prognostication in Intensive Care Unit and Emergency Room
LOS: Length of hospital stay
PCO_2_: Arterial carbon dioxide
PSM: Propensity score-matched
qSOFA: Quick sepsis-related organ failure assessment
VFD: Ventilator-free days
