# Supplementary material for: In-hospital mortality associated with the misdiagnosis or unidentified site of infection at admission
Source: Crit Care. 2019 Jun 6;23:202. doi: 10.1186/s13054-019-2475-9 (PMC6551884; doi:10.1186/s13054-019-2475-9)
Supplement: Supplementary file 1 — Table S1. Baseline characteristics of patients with infection in the propensity-matched model. BMI = body mass index, GCS = Glasgow coma scale, MBP = mean blood pressure, HR = heart rate, RR = respiratory rate, ref. = reference, the SD = standardized difference. Rare: central nervous system (CNS); osteoarticular; endocardium; wound; catheter-related; and implant device-related at final diagnosis. (DOCX 20 kb) [file 13054_2019_2475_MOESM1_ESM.docx]

Table S1: Baseline characteristics of patients with infection in the propensity-matched model

| Characteristics | | Misdiagnosis or unidentified | Correct | SD (%) |
| --- | --- | --- | --- | --- |
|  | | 77 | 77 |  |
| Age at admission (years old) | | 79 (68-86) | 77 (66-85) | 8.7 |
| Charlson comorbidity index | | 1 (0–2) | 1 (0–3) | −7.3 |
| Clinical frailty scale | | 4 (2–7) | 4 (3–6) | 11.3 |
| GCS | | 14 (11–15) | 14 (11–15) | −1.2 |
| MBP (mmHg) | | 92 (75–108) | 91 (75–103) | 6.9 |
| HR (/min) | | 100 (78–115) | 103 (84–111) | −11.3 |
| RR (/min) | | 23 (18–26) | 21 (18–24) | 13.4 |
| Site of infection  at the final diagnosis | Lung | 23 (29.9) | 20 (26.0) | ref |
|  | Intra-abdominal | 13 (16.9) | 10 (13.0) | 9.9 |
|  | Urinary tract | 20 (26.0) | 26 (33.8) | −19.5 |
|  | Soft tissue | 6 (7.8) | 9 (11.7) | −16 |
|  | Rare | 7 (9.1) | 5 (6.5) | 11.2 |
|  | Other | 8 (10.4) | 7 (9.1) | 5 |
|  | Unidentified | 0 (0) | 0 (0) | - |
| BMI = body mass index, GCS = Glasgow coma scale, MBP = mean blood pressure, HR = heart rate, RR = respiratory rate, ref = reference, the SD = standardized difference Rare: central nervous system (CNS); osteoarticular; endocardium; wound; catheter-related; and implant device-related at a final diagnosis. | | | | |
